# The Distribution and Origins of *Pyrus hopeiensis-*“Wild Plant With Tiny Population” Using Whole Genome Resequencing

**DOI:** 10.3389/fpls.2021.668796

**Published:** 2021-06-17

**Authors:** Yongtan Li, Jun Zhang, Shijie Wang, Yiwen Zhang, Minsheng Yang

**Affiliations:** ^1^Forest Department, Forestry College, Hebei Agricultural University, Baoding, China; ^2^Hebei Key Laboratory for Tree Genetic Resources and Forest Protection, Baoding, China

**Keywords:** *Pyrus hopeiensis*, SNPs, whole genome re-sequencing, PCA, Fst and π ratio, KEGG

## Abstract

*Pyrus hopeiensis* is a valuable but endangered wild resource in the genus *Pyrus*. It has been listed as one of the 120 wild species with tiny population in China. The specie has been little studied. A preliminary study of propagation modes in *P. hopeiensis* was performed through seed propagation, hybridization, self-crossing trials, bud grafting, branch grafting, and investigations of natural growth. The results showed that the population size of *P. hopeiensis* was very small, the distribution range was limited, and the habitat was extremely degraded. In the wild population, natural hybridization and root tiller production were the major modes of propagation. Whole genome re-sequencing of the 23 wild and cultivated accessions from *Pyrus* species collected was performed using an Illumina HiSeq sequencing platform. The sequencing depth range was 26.56x−44.85x and the average sequencing depth was 32x. Phylogenetic tree and principal component analyses (PCA) based on SNPs showed that the wild *Pyrus* species, such as PWH06, PWH07, PWH09, PWH10, PWH13, and PWH17, were closely related to both *P. hopeiensis* HB-1 and *P. hopeiensis* HB-2. Using these results in combination with morphological characteristics, it speculated that *P. hopeiensis* populations may form a natural hybrid group with frequent gene exchanges between and within groups. A selective elimination analysis on the *P. hopeiensis* population were performed using Fst and π radio and a total of 381 overlapping genes including *SAUR72, IAA20, HSFA2*, and *RKP* genes were obtained. These genes were analyzed by gene ontology (GO) and Kyoto Encyclopedia of Genes and Genomes (KEGG) function enrichment. And four KEGG pathways, including lysine degradation, sphingolipid metabolism, other glycan degradation, and betaine biosynthesis were significantly enriched in the *P. hopeiensis* population. Our study provided information on genetic variation, evolutionary relationships, and gene enrichment in *P. hopeiensis* population. These data will help reveal the evolutionary history and origin of *P. hopeiensis* and provide guidelines for subsequent research on the locations of functional genes.

## Introduction

*Pyrus hopeiensis* (2n = 34), belonging to *Pyrus*, is a rare wild resource of the genus that is unique to China. It is distributed in the provinces of Hebei and Shandong. Because of its limited distribution and population declines, *P. hopeiensis* has been classified as “the wild plants with tiny population” (Li et al., 2018) that urgently requires protection. It is an arbor. The leaves are ovate, broadly ovate to almost round, with long or short acuminate apices. The white flowers are clustered in umbrella-shaped racemes. Fruits are brown and generally have four ventricles, rarely five. The fruit has multiple lenticels and stone cells. The flowering period is during April, and the fruiting period is from September to October. Pu et al. (1985) also regarded *P. hopeiensis* as a separate species based on karyotype. Jiang et al. (1992) divided the genus *Pyrus* into 14 species, including *P. hopeiensis*, which was recognized by morphological characteristics. Based on the external morphology of the species, Yu (1984) suggested that it may have originated as a natural hybrid between *Pyrus ussuriensis* and *Pyrus phaeocarpa* and it is secondary species in the evolutionary process. However, the evolutionary origins of the species require further study.

Pears have significant phenotypic variation. Further studies of the genetic diversity of pear, and the identification of interesting gene-controlling traits will be key elements in future pear genetic breeding programs. However, pear is self-incompatible, and has a complex genetic background, a highly heterozygous genome, and a long breeding cycle, which make it difficult for breeders to directly determine phenotype-genotype relationships (Wang et al., 2016; Zhang et al., 2018). Moreover, it is easy to hybridize between *Pyrus* species, and the differences of biological and morphological characters between species or varieties are not obvious, which greatly increases the difficulty of phylogenetic evolution and classification of pears. The whole genome resequencing is to resequence the genomes of different individuals or populations of species with existing reference genomes in order to detected a large number of genetic variation information including SNP, Indel, SV, CNV etc. With the continuing publication of the pear genome (Wu et al., 2013; Linsmith et al., 2019; Ou et al., 2019; Dong et al., 2020) and the rapid development of high-throughput sequencing technologies, it provided a foundation for a genome-wide variation analysis of germplasm resources and the functional gene mining, phylogenetic analysis, population genetic structure, genetic diversity, analyses and selective elimination (Zhou et al., 2020), which is of great significance for the study of molecular breeding and population evolution of species. Li et al. (2020) used whole-genome resequencing to analyze the evolution and domestication characteristics of 69 lotus accessions founding that the flower lotus was biphyletic and genetically heterogeneous, whereas the rhizome and seed lotus were monophyletic and genetically homogeneous. Besides they identified a total of 1,346 selected regions in different lotus. Further analysis showed that genes in these regions were related to the important domestication traits of lotus such as insect resistance, seed weight, size and nutritional quality, freezing, and heat stress resistance.

To date, there are few reports on *P. hopeiensis*. The number, distribution, origins, and evolution of wild *P. hopeiensis* trees remain unclear. Information on the unique adaptability and genetic responses of *P. hopeiensis* to environmental pressure is lacking, and little is known of adaptation and genetic control of survival in natural habitats. In this study, we conducted a preliminary investigation about the quantity, distribution, natural environment, and reproductive mechanisms of *P. hopeiensis* and performed the first whole genome re-sequencing of 23 *Pyrus* accessions and carried out a detailed study of genomic mutation sites. And the SNPs obtained were used for population structure analysis of *P. hopeiensis* and other pears. Through a selective elimination analysis, the selection area of wild *P. hopeiensis* under natural conditions were accurately screened. At the same time, Gene ontology (GO) and Kyoto Encyclopedia of Genes and Genomes (KEGG) function enrichment analysis of the screened genes were also performed. Our analyses will improve understanding of the selection pressures on *P. hopeiensis* during the evolutionary process and will provide a basis for the effective use of *P. hopeiensis* resources.

## Materials and Methods

### Sample Collection and Genomic DNA Extraction

**Figure 1:**
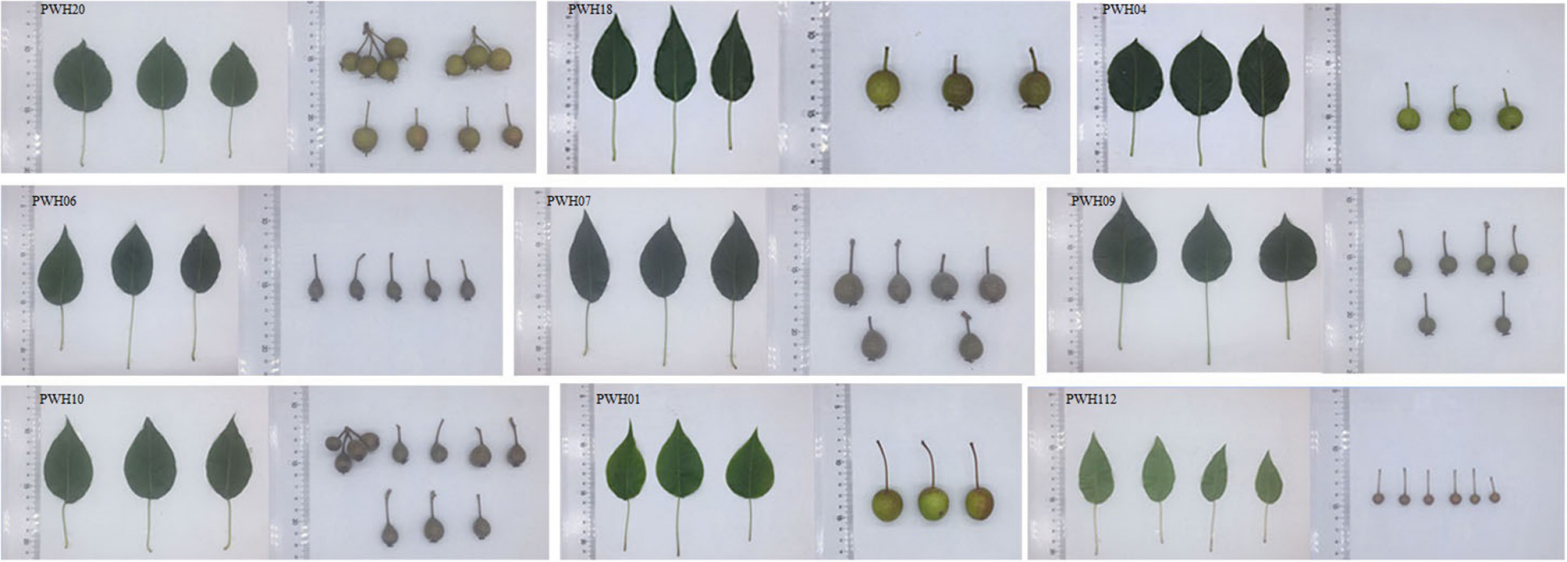
Images of leaves and fruit of the nine wild pears (PWH01, PWH04, PWH06, PWH07, PWH09, PWH10, PWH12, PWH18, PWH20) included in this study.

In 2019, a survey of *P. hopeiensis* populations and other wild resources in Xingshuyuan and on Changyushan in Changli, Hebei Province were conducted. And a total of 20 wild *Pyrus* accessions and three locally cultivated *Pyrus* accessions were collected (Supplementary Table 1). The samples included *Pyrus hopeiensis* HB-1 (*n* = 3), *Pyrus hopeiensis* HB-2 (*n* = 2), *Pyrus bretschneideri* 'Guali' (*n* = 3), *Pyrus betulifolia* (*n* = 2), *P*yrus *ussuriensis* Maxin. cv. Jingbaili (*n* = 1), *Pyruscommunis* L. cv. Early Red Comice (*n* = 1), and other wild pears (*n* = 11). The leaf and fruit morphologies of part of the wild plants collected were shown in Figure 1. The total DNA from fresh young leaves of the 23 *Pyrus* accessions were extracted using plant DNA extraction kit (TIANGEN, Beijing). And the extracts were sent to Novogene Co., Ltd. (Beijing) for whole genome re-sequencing. Agarose gel electrophoresis was used to analyze the RNA quality, degradation and contamination. The NanoDrop spectrophotometer (Thermofisher, Shanghai) was used to determine the purity of the DNA, and Qubit fluorometry (Thermofisher) was used to accurately quantify the DNA concentrations. Finally, the optical density (OD) values of DNA ranged between 1.8 and 2.0 and the content above 1.5 ug were used to build the library.

### Pollination Tests

Cross-pollination and self-pollination (Supplementary Table 2) trials were performed at the end of March and in early April 2017 and 2018. The anthers were collected 1 day before flowering and the pollen were collected for pollination after dispersal at indoor. For self-pollination tests, two or three lateral flowers were selected for bagging before flowering and the other flowers were picked. Moreover, the artificial pollination was also performed. In the cross-pollination trials, the androecia of the flowers were removed 1 day before pollination and the flowers were bagged after pollination.

### Library Construction and Whole Genome Re-sequencing

The qualified DNA samples were randomly broken into fragments of 350 bp in length using a Covaris (Guangzhou) ultra-sonic shearer. A Truseq Library Construction Kit (Illumina, Shanghai) was used to build the library. The entire library was prepared in the following steps: end repair, polyA tail addition, sequencing connector addition, purification, PCR amplification. Then the constructed library was sequenced with an Illumina HiSeq sequencing system. To obtain clean data, the low quality paired reads were removed based on the following criteria: reads with adapters, paired reads with N content exceeding 10% of the length of the read and low-quality (Q ≤ 5) paired reads contained in single-end sequencing reads that exceeded 50% of the length of the read.

### Variant Detection and Annotation

The effective sequencing data were compared with the reference genome of *Pyrus communis* Bartlett DH Genome v2.0 (https://www.rosaceae.org/species/pyrus/pyrus_communis/genome_v2.0) using BWA (Li and Durbin, 2009) software (parameter: mem -t 4 -k 32 -M). SAMtools (Li et al., 2009) and Picard (http://picard.sourceforge.net) software were used to remove duplicates.

SAMtools software was used to detect the SNPs and insertion-deletion mutations (InDels) in 23 *Pyrus* accessions and ANNOVAR software was used for annotation (Wang et al., 2010). To reduce the detection error rate, SNPs with quality values (MQ) <20 and SNPs with read support numbers <4 were filtered out. The snpEff software (https://pcingola.github.io/SnpEff/) was used to annotate the filtered SNPs based on the *Pyrus communis* Bartlett DH Genome v2.0 genome. And the SNPs were classified according to their impact. Meanwhile, the insertion and deletion of small fragments <50 bp in length were detected.

Breakdancer (Chen et al., 2009) and CNVnator software (Abyzov et al., 2011) were used to detect structure variants (SVs) and copy number variants (CNVs), respectively. Breakdancer software was used to detect insertion (INS), deletion (DEL), inversion (inversion, INV), intra-chromosomal translocation (ITX), and inter-chromosomal translocation (CTX). The SVs with support numbers of paired-end (PE) reads <2 were filtered out. CNVnator was used to detect different read coverage depths on the genome to determine potential deletion and duplication. Then ANNOVAR was used to perform functional annotations of the all gene mutations detected. Finally, the circos (Krzywinski et al., 2009) were used to visualize the structural variation in *P. hopeiensis*.

### Population Structure Analysis

A neighbor-joining tree was constructed using TreeBeST 1.9.2 software (Jin et al., 2012) with 1000 bootstrap replicates based on SNPs detected in whole genome resequencing analysis. Principal component analysis (PCA) was performed using gcta64 1.92.2 software (Yang et al., 2011). Population clustering analysis were calculated by ADMIXTURE 1.3.0 software (Alexander et al., 2009). The number of clusters was varied from *K* = 2 to *K* = 9 and the lowest cross-validation error was used to determine the optimal K value in order to determine the ideal population structure.

### Selective Elimination Analysis

In order to detect the selected regions of *P. hopeiensis*, and to determine the identities of the selected genes to explain the adaptation mechanism in population evolution and domestication at the molecular level, PopGenome software (Pfeifer et al., 2014) was used to perform a sliding window according to physical length (100 kb as the window and 10 kb as the step size) based on the filtered high-quality SNPs to analyzed the π ratio (Lin et al., 2014) and the genetic differentiation index (Fst) (Hudson et al., 1992) between the two populations. At the same time, the significant regions were screened by the top 5% to identified the genes in these selected regions based on the Fst and π ratio analyses. And the Fst and π ratio values were combined to obtain the candidate genes in the selected region and to complete the construction of the Venn diagram. All relevant charts were plotted by R scripts (Team, 2013).

### Variant Functional Annotation and Enrichment Analysis

GO and KEGG pathway enrichment analyses were conducted on the selected area of the genome. The numbers of genes enriched in different GO entries were counted and the GO entries significantly enriched in the candidate region were determined. Besides, the KEGG enrichment analysis was also performed to identify the significantly enriched pathways. The enriched pathways and genes were selected stringently using an adjusted probability (*P* < 0.05).

## Results and Analysis

### Distribution and Habitats of *Pyrus hopeiensis*

**Figure 2:**
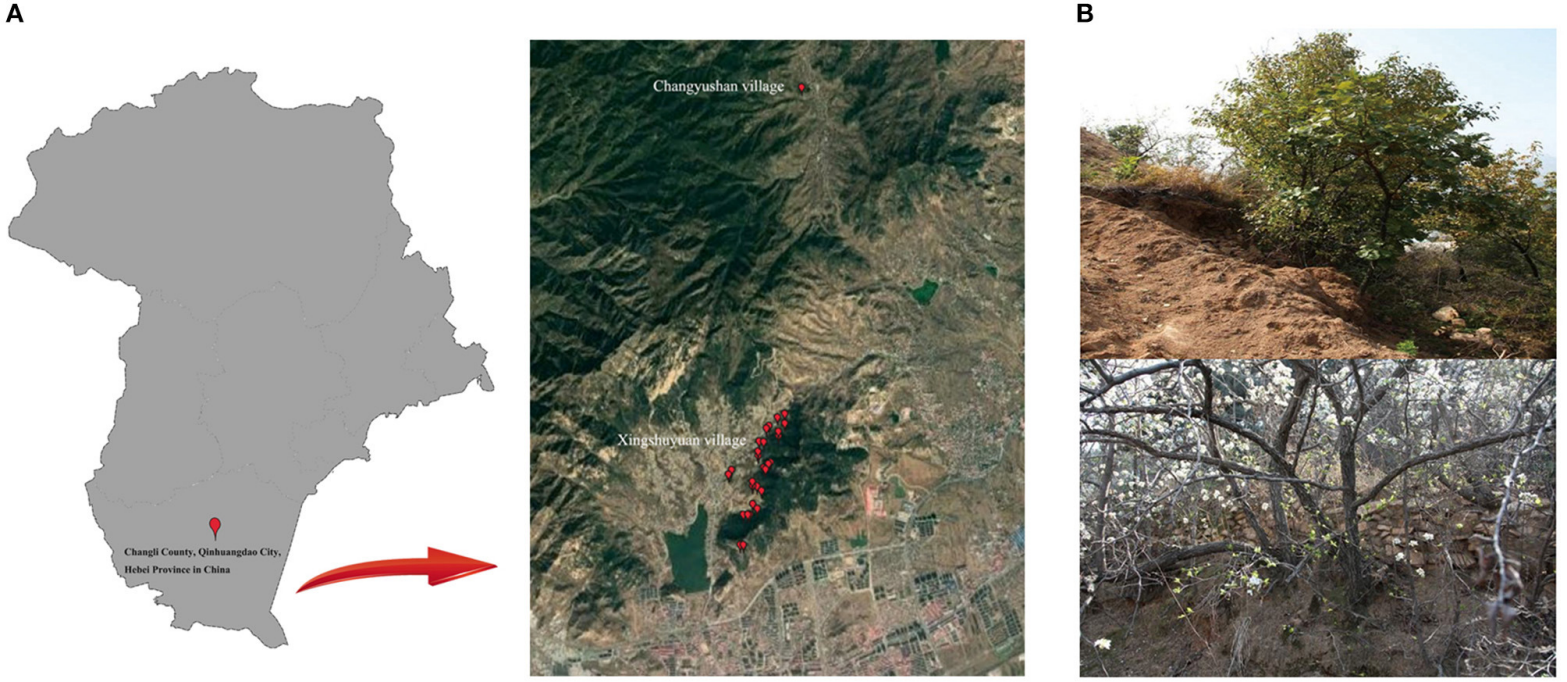
**(A)** Distribution of part of the *P*y*rus hopeiensis*; **(B)** Habitat of *Pyrus hopeiensis*.

At present, the *P. hopeiensis* were found only in Changli, Hebei Province and Laoshan, Shandong Province. Between 2016 and 2019, multiple surveys of the numbers, habitats and distribution of the species in Changli were conducted. The surveys showed that *P. hopeiensis* had disappeared in many places; the trees were found only in Xingshuyuan and Changyushan village (Figure 2A). Based on phenotype and previous SSR analysis, it initially believed that two main types of *P. hopeiensis*r existed: *P. hopeiensis* HB-1 and *P. hopeiensis* HB-2. The *P. hopeiensis* HB-1 population occurred mainly in Xingshuyuan. More than 100 *P. hopeiensis* specimens were found, most of which were shrubs generated by root sprouting. And only 10 tree form specimens remained. The maximum age of the individuals was *ca*. 50 years. In the early stage of our work, some individuals with SSR molecular markers were tested and no differences were found among these specimens. The *P. hopeiensis* HB-2 populations found only five strains were distributed on Changyushan. After verification using SSR molecular markers, no differences were found among the individuals. The *P. hopeiensis* populations lived under very harsh conditions (Figure 2B). They grew mostly on cliffs and had bare roots. As the fruits of *P. hopeiensis* are small and inedible, local people mostly used the trees as rootstocks or felled them to provide new areas for development. So few *P. hopeiensis* individuals survived.

### Preliminary Study of the Reproduction Mode in *Pyrus hopeiensis*

**Figure 3:**
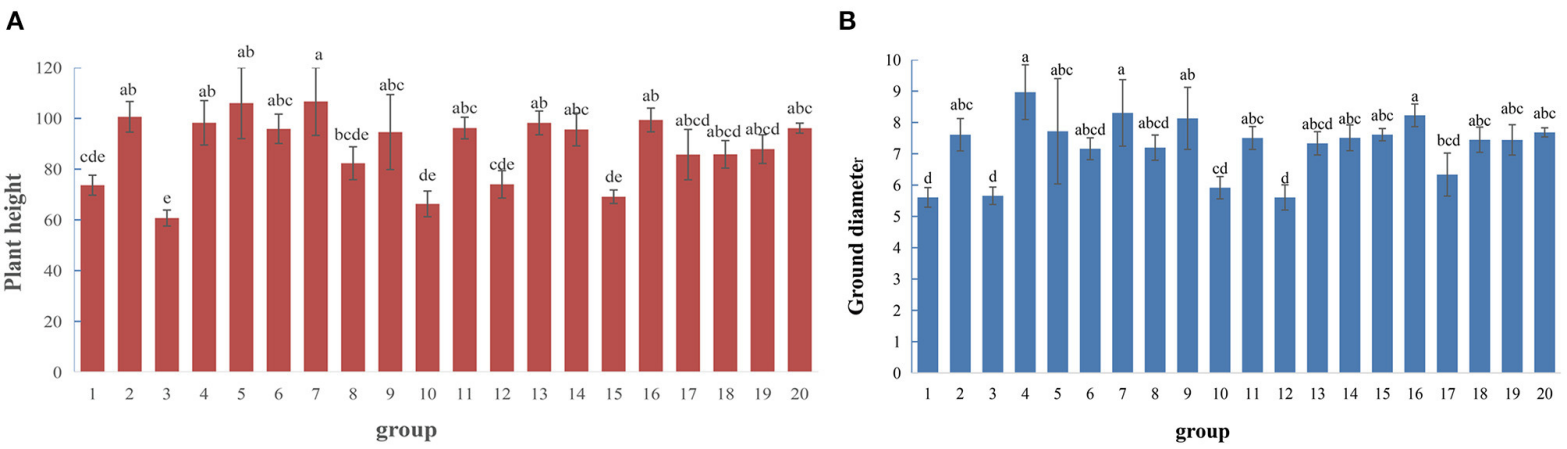
Plant height **(A)** and ground diameter **(B)** of the hybrid combination.

Using *P. hopeiensis* individuals (No. 1 and No. 5) as the female parents, and the five cultivated pear strains (Huangguan, Yali, Weixian red pear, Xuehua, and Dunzi pear) as the male parents, the hybridization experiments were performed and a total of 1,190 hybrid seeds were obtained. When *P. hopeiensis* were used as the male parent and Xianghong and Yali as the female parents, these crosses produced a total of 281 hybrid seeds (Supplementary Table 2). The plant height and ground diameter of the offspring plants from each cross (now planted in the Changli Institute for Pomology) were measured. The average height of progeny plants from hybrid combination 7 (No. 5 *P. hopeiensis* HB-1 × Yali) was 106.63 cm, the highest among crosses (Figure 3A). The progeny plants of hybrid combination 4 (No. 1 *P. hopeiensis* HB-1 × Xue hua) had the largest average ground diameter (8.9 cm) (Figure 3B). The plant height and ground diameter varied among different offspring. The results showed that the progeny of different cross combinations were quite different. The progeny of Yali and *P. hopeiensis* had greater average plant heights and ground diameters than the progeny of Xianghong and *P. hopeiensis*. In total, 1,839 natural hybrid seeds collected of *P. hopeiensis*. After sand storage, accelerated budding and sowing, 466 progeny plants of *P. hopeiensis* were obtained in the following year. These individuals were planted at the Changli Institute for Pomology, where they were growing well. The plant heights ranged between 69.11 and 99.37 cm (Figure 3A), and the ground diameter ranged between 6.34 and 8.23 cm (Figure 3B).

The bagging for self-crossing of *P. hopeiensis* in Xingshuyuan obtained no fruits. Thus, it provisionally categorized *P. hopeiensis* as self-incompatible. The different *P. hopeiensis* plants were cross pollinated and the same individuals were self-pollinated (Supplementary Table 2). Again, no fruits were produced indicating that *P. hopeiensis* was self-incompatible.

The stems of sterile seedlings of *P. hopeiensis* were used as source materials for culture. A medium was optimized to establish tissue cultures and a rapid propagation system for *P. hopeiensis* and 1,000 rooted tissue culture seedlings were obtained. In addition, 80 *P. hopeiensis* seedlings obtained by bud grafting grew well. The one-year-old branches of *P. hopeiensis* as scions, and 1- or 2-year-old seedlings as rootstocks were collected for grafting and propagation. A total of 2,200 *P. hopeiensis* seedlings were obtained and 1,900 of these were provided to diverse *ex-situ* conservation bases.

The investigation of the natural propagation mode of *P. hopeiensis* showed that individuals at some sites were clustered. We excavated the roots and found that the specimens were derived from root tillers, and no seedlings were found. An earlier SSR molecular marker detection investigation showed that *P. hopeiensis* trees occurring in Xingshuyuan belonged to a single genotype and had low genetic diversity. Thus, it came to the provisional conclusion that *P. hopeiensis* reproduced mainly by root tillers under natural conditions.

In summary, it postulated that *P. hopeiensis* could be bred through hybridization, tissue culture, bud grafting, branch grafting and propagation by root tillers. Moreover, it was self incompatible but propagated through root tillers in its natural environment.

### Variation in the *Pyrus* Genome

The sequencing produced 399.361G raw data from 23 *Pyrus* accessions, and 388.774G clean data after filtering. The sequencing quality was high (Q20 ≥ 96.35%, Q30 ≥ 90.36%) (Supplementary Table 3). Paired-end sequencing reads were mapped to the reference genome (*P*. *communis* Bartlett DH Genome v2.0). The reference genome size was 498,265,991 bp and the GC content (%) was 37.47%.

The comparison ratio of all samples were between 92.75 and 98.15%, the coverage depth was between 26.56x and 44.85x, and the 1X coverage (with at least one base coverage) was >85.95% (Supplementary Table 4). The comparison result was normal and suitable for subsequent mutation detection and related analyses.

**Figure 4:**
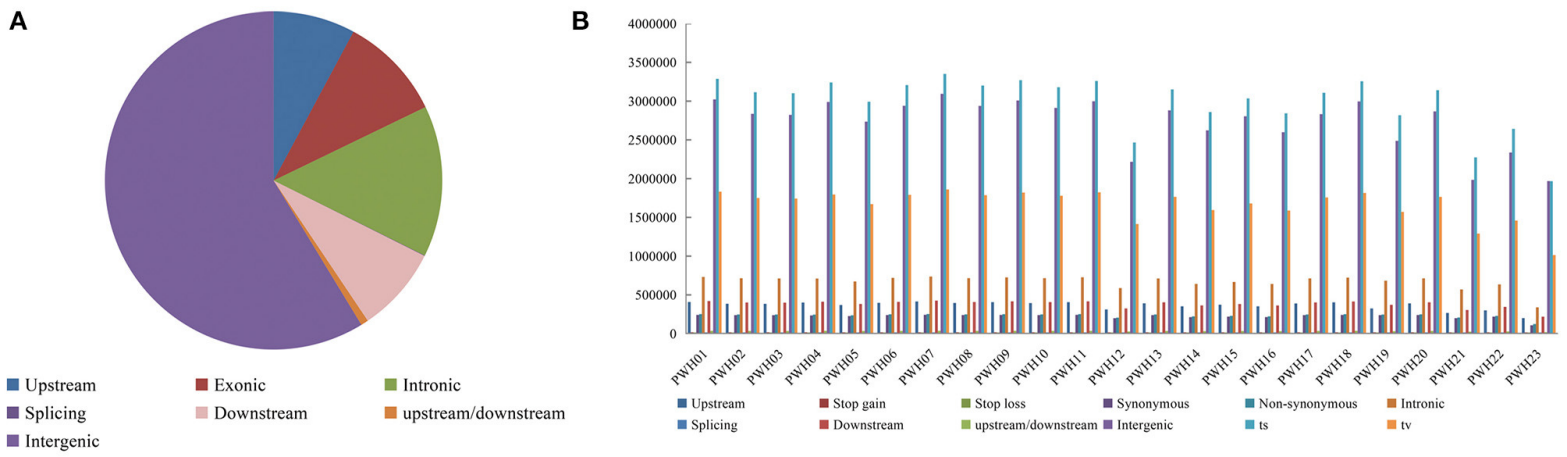
**(A)** Distribution of SNPs in genomic regions. **(B)** Number of different SNPs in genomic regions per species.

The genome-wide variations in 23 *Pyrus* accessions included 107,133,072 single nucleotide polymorphisms (SNPs), 14,186,626 insertions or deletions (InDels), 290,443 copy number variations (CNVs), and 1,317,828 structural variations (SVs). The numbers of SNPs detected ranged from 2,980,930 (*P*. *communi* L. cv. Early Red Comic) to 5,211,026 (PWH07). Most mutation sites were located in non-coding regions (Table 1), such as intron and intergenic regions. Among them, 58.71% of SNPs were located in intergenic regions, 14.48% of SNPs were located in intron regions, and 9.98% were located in coding regions (Figure 4A). The SNP in coding regions included 107,289–242,631 synonymous substitutions and 124,051–253,183 non-synonymous substitutions (Figure 4B), giving non-synonymous-to synonymous substitution ratios (dN/dS) in the range 1.030–1.156 showing that the numbers of non-synonymous mutations exceeded the numbers of synonymous mutations. The transition/transversion (ts/tv) ratio values were in the range 1.743–1.939 and the heterozygosity rate of different *Pyrus* accessions varied greatly, ranging from 0.19 to 0.53%, with an average of 0.44%. Based on the type of change and its predicted effect, 1.53% of the SNPs were predicted to have high impacts (e.g., stop codon gaining, frameshift), 1.38% moderate impacts (e.g., non-synonymous change, non-disruptive frameshift), and 0.15% low impacts (e.g., synonymous coding/start/stop, start gained).

**Table 1:** Genome-wide variations identified in 23 *pyrus* accessions.

**Sample**	**SNP**				**InDel**					**SVs**			**CNVs**	
	**Exonic**	**Intronic**	**Intergenic**	**Ts/Tv**	**Intronic**	**Intergenic**	**Exonic**	**Insertion**	**Deletion**	**Insertion**	**Deletion**	**Inversion**	**Duplication**	**Deletion**
PWH01	497,407	731,695	3,022,946	1.794	108,084	424,445	21,003	311,993	382,637	8	30,523	3,987	2,257	13,699
PWH02	490,142	714,771	2,836,382	1.779	103,383	388,347	20,397	287,882	355,816	16	29,013	2,839	2,543	10,437
PWH03	488,846	712,124	2,822,707	1.779	104,231	393,575	20,426	291,584	359,736	3	29,383	2,931	2,329	11,197
PWH04	483,456	710,758	2,989,293	1.805	104,096	411,415	20,465	303,275	369,504	16	29,446	3,413	2,187	11,836
PWH05	466,819	673,856	2,735,263	1.790	99,476	378,858	19,718	281,958	343,907	4	27,601	2,973	2,601	10,203
PWH06	493,236	719,447	2,940,119	1.790	104,932	402,299	20,356	297,537	365,031	13	29,348	2,935	2,515	9,864
PWH07	500,086	736,339	3,094,414	1.801	107,837	424,419	20,784	312,314	381,694	9	30,294	3,577	2,349	11,007
PWH08	492,085	715,896	2,938,303	1.791	104,339	401,287	20,090	296,653	363,435	17	28,985	2,909	2,564	9,803
PWH09	495,960	725,608	3,007,952	1.796	106,480	415,733	20,591	306,508	375,004	11	29,705	3,305	2,414	10,844
PWH10	491,209	715,918	2,912,122	1.786	104,271	398,482	20,192	294,918	361,608	14	28,313	2,586	2,493	9,719
PWH11	497,550	727,125	2,998,096	1.787	105,912	409,714	20,575	300,818	372,886	18	29,507	3,046	2,475	10,039
PWH12	408,575	587,976	2,217,100	1.743	92,748	334,394	18,406	253,708	307,715	14	26,076	2,520	2,082	11,969
PWH13	490,300	712,078	2,880,922	1.785	103,774	395,558	20,232	292,948	359,891	17	28,531	2,658	2,483	9,692
PWH14	439,527	641,621	2,622,574	1.793	95,950	368,686	19,100	274,988	331,822	18	26,911	2,640	2,301	11,583
PWH15	452,739	667,052	2,804,306	1.806	99,181	388,402	19,355	287,066	348,477	8	28,635	3,140	2,358	11,095
PWH16	440,660	640,828	2,598,913	1.790	96,097	369,910	19,107	275,981	332,957	33	27,996	2,809	2,431	10,527
PWH17	490,064	713,038	2,832,335	1.768	105,475	399,815	20,410	296,482	364,472	31	29,143	3,021	2,288	11,091
PWH18	495,818	723,160	2,995,871	1.794	105,950	413,020	20,444	304,686	372,328	11	29,598	3,153	2,322	10,754
PWH19	488,799	683,064	2,487,286	1.793	94,456	301,823	19,327	230,503	293,330	0	18,892	2,073	5,851	3,573
PWH20	492,667	713,610	2,867,136	1.780	104,043	393,495	20,367	290,150	361,238	7	28,752	2,694	2,519	9,823
PWH21	410,837	570,281	1,984,188	1.761	84,602	261,906	17,648	204,792	255,526	0	17,889	1,945	5,874	3,962
PWH22	452,133	635,340	2,337,311	1.810	88,530	282,245	18,394	216,520	274,114	0	19,303	2,178	5,268	3,082
PWH23	233,972	338,958	1,968,681	1.939	48,061	215,160	13,405	142,262	197,972	0	11,924	2,860	8,269	3,871

*Exonic, variants located in an exonic region; Intronic, variants located in an intronic region; Intergenic, variants located in an intergenic region; ts, transition; tv, transversion*.

**Figure 5:**
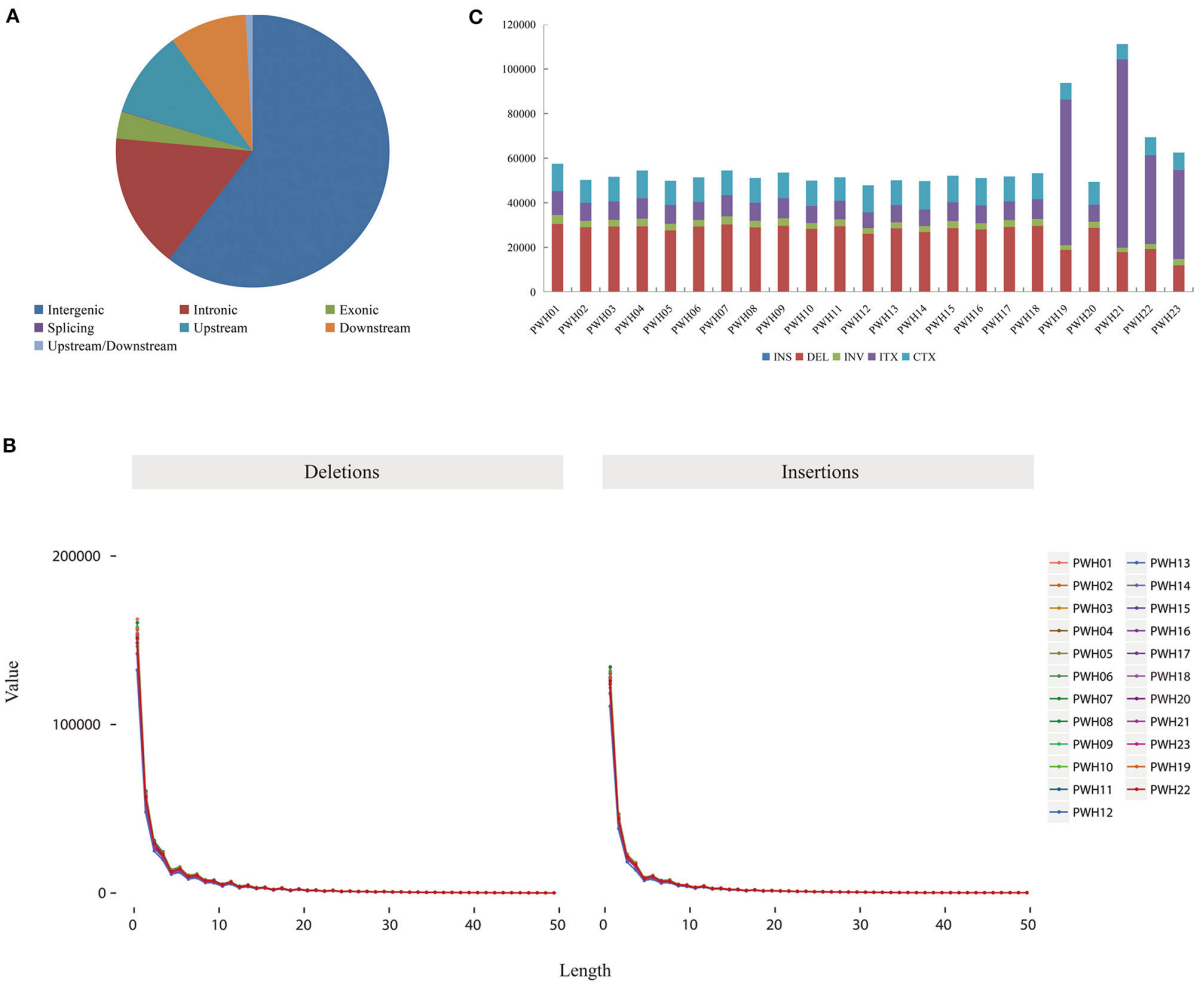
**(A)** Distribution of SNPs in genomic regions. **(B)** The distribution of small insertions and deletions in genomic regions per species. **(C)** The number of different SVs in genomic regions per species.

Regarding InDels, the number of InDels mutations detected ranged from 340,234 (*P*. *communis* L. cv. Early Red Comic) to 694,630 (PWH01). And the total number of InDel variants amounted to 14,186,626, of which 6,355,526 were insertions and 7,831,100 deletions showing that the numbers of deletions exceeded the insertions. Besides, the Indel heterozygous rates of different *Pyrus* accessions differed, ranging from 0.16 to 0.47‰ (average 0.39‰). Among those with potential functional consequences, 3.18% were located in gene exons and 0.077% were located in splice site regions. What is more, 60.43% (8,572,988) were distributed in the intergenic region, 16.01% (2,271,908) were distributed in the intron region, and 3.18% (18,046) were distributed in the coding region among all the detected Indels. And the vast majority of Indels were located upstream or downstream from genes and intergenic regions (Figure 5A). Moreover, the lengths of insertions and deletions were in the range 1–50 nucleotides (Figure 5B) (Supplementary Tables 5, 6).

**Figure 6:**
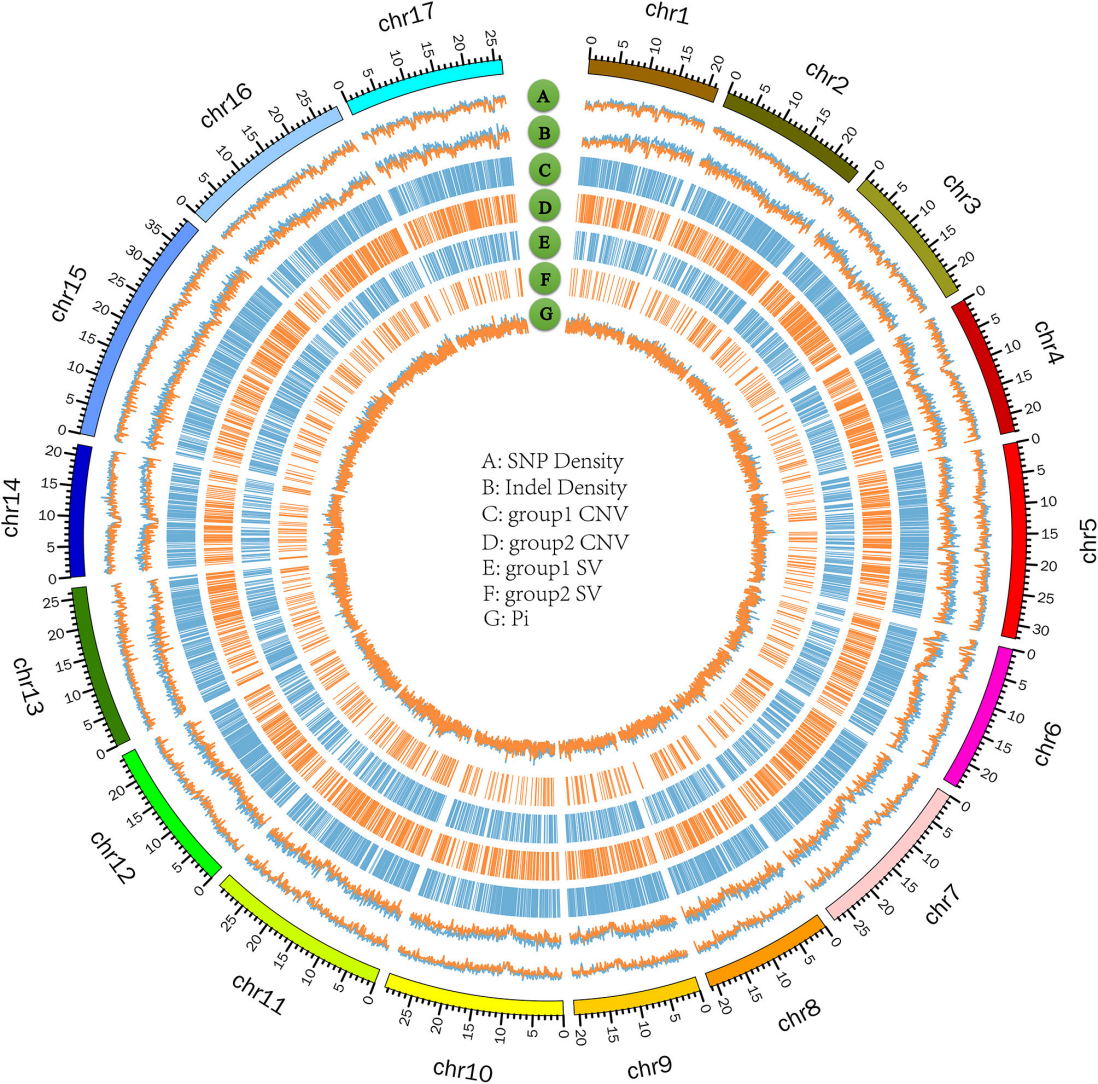
Circos plot on 17 chromosomes of *P. hopeiensis*. **(A)** SNP density; blue refers to group 1, orange to group 2. **(B)** Indel density; blue refers to group 1, orange to group 2. **(C,D)** CNV in group 1 and group 2, including deletion and duplication. **(E,F)** SV in group 1 and group 2, including insertion and deletion. **(G)** Average number of nucleotide differences per site (pi); blue refers to group 1, orange to group 2. Group 1 contains *P. hopeiensis* HB-2, group 2 contains *P. hopeiensis* HB-1.

The numbers of SVs among the 23 *Pyrus* accessions ranged between 47,827 (PWH12) and 111,172 (PWH21) (Figure 5C). The deletions, insertions, inversions, ITX, and CTX accounted for 46.73% (615,768), 0.02% (268), 5.02% (6,6192), 29.55% (389,468), and 18.68% (246,132) of the detected SVs, respectively. And the CNVs ranged from 8,350 (*P*. *ussuriensis* Maxin. cv. Jingbaili) to 15,956 (PWH01), with an average of 12,628. Besides, the number of duplications ranged from 2,082 (PWH12) to 8,269 (*P*. *communis* L. cv. Early Red Comice) and the number of deletions ranged from 3,082 (*P*. *ussuriensis* Maxin. cv. Jingbaili) to 1,3699 (PWH01). Among these *Pyrus* species, 34,867 CNV and 198,137 SV were detected in *P. hopeiensis* HB-1, and 24,856 CNV and 100,806 SV were detected in *P. hopeiensis* HB-2, respectively (Figure 6).

### Population Structure of *Pyrus*

**Figure 7:**
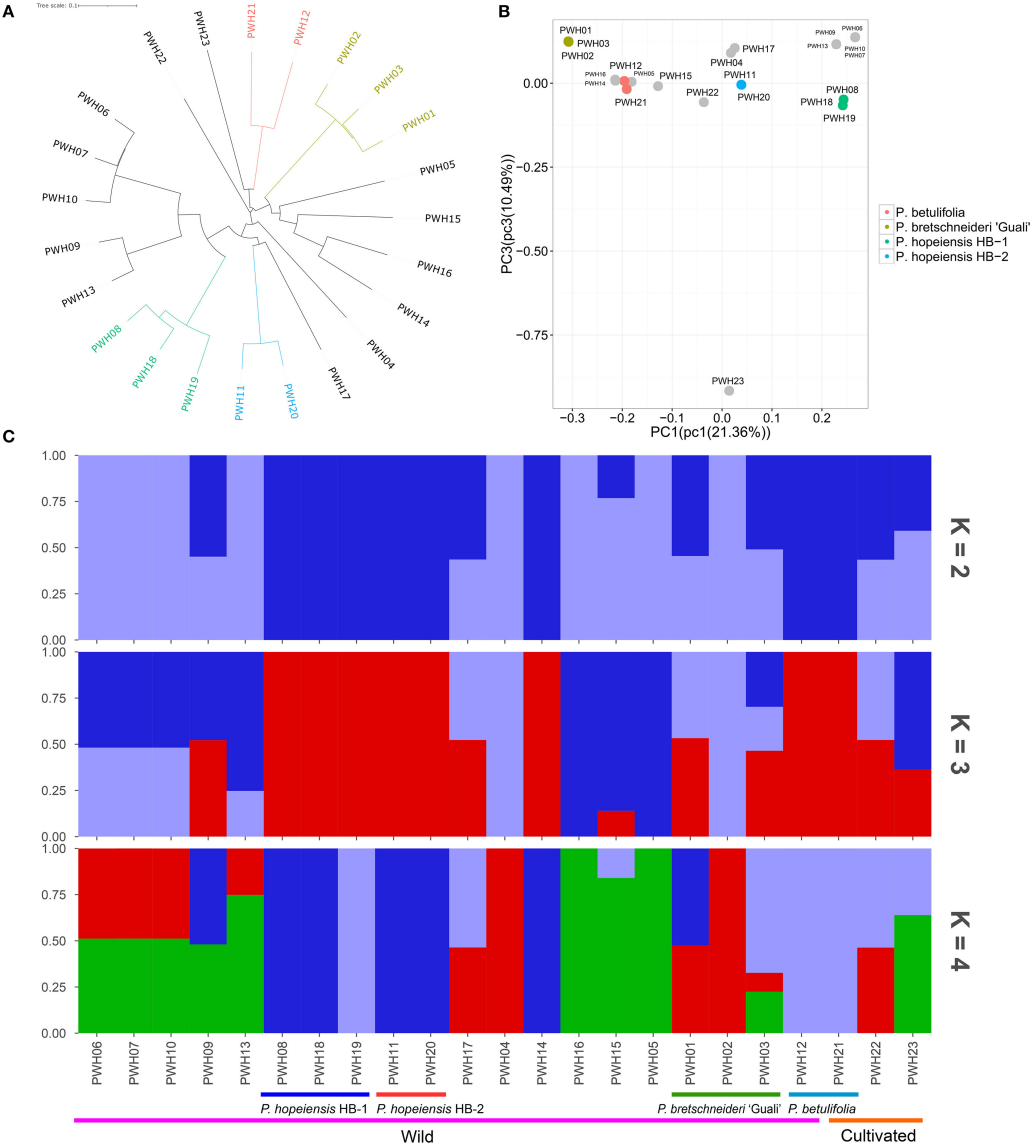
Population structure. **(A)** Neighbor-joining phylogenetic tree constructed using SNP data. **(B)** Principal component analysis (PCA) of the 23 *Pyrus* accessions. **(C)** Population structure (*K* = 2–4) of the 23 *Pyrus* accessions.

After filtering, the high-quality SNPs identified on 17 chromosomes (Supplementary Figure 1) were used as markers in the subsequent principal component analysis (PCA), population structure and evolutionary tree analysis. The neighbor-joining tree showed that the *P. hopeiensis* populations were arrayed on two different branches (Figure 7A). The *P. hopeiensis* HB-1 population was on one branch, and the *P. hopeiensis* HB-2 population on the other branch. Two *P*. *betulifolia* plotted together, and three *P*. *bretschneideri* ‘Guali' plotted together. Moreover, PWH06, PWH07, PWH09, PWH10, PWH13 wild pears, and *P. hopeiensis* HB-1 were close to one another on the neighbor-joining tree. PWH17 and *P. hopeiensis* HB-2 were close. The PCA separated individuals with different genetic backgrounds and grouped individuals with similar genetic backgrounds. The PCA results was similar to that of the neighbor-joining tree. Individuals of *P. hopeiensis* HB-1 and *P. hopeiensis* HB-2 were clustered together in the PCA plot (Figure 7B). The population structure showed that the cross-validation error rate was smallest when the kinship coefficient (*K*) = 3 (Supplementary Figure 2). And when *K* = 3, the genetic backgrounds between *P. hopeiensis* HB-1 and *P. hopeiensis* HB-2 populations were identical and the genes penetration between PWH01, PWH03, PWH09, PWH12, PWH14, PWH15, PWH17, PWH21, PWH22, PWH23, and *P. hopeiensis* were found (Figure 7C). The results that were not fully congruent with the neighbor-joining tree and PCA plots. Supplementary Figure 3 showed the outcomes when *K*= 5–9.

### Identification of Positive Selective Sweeps

**Figure 8:**
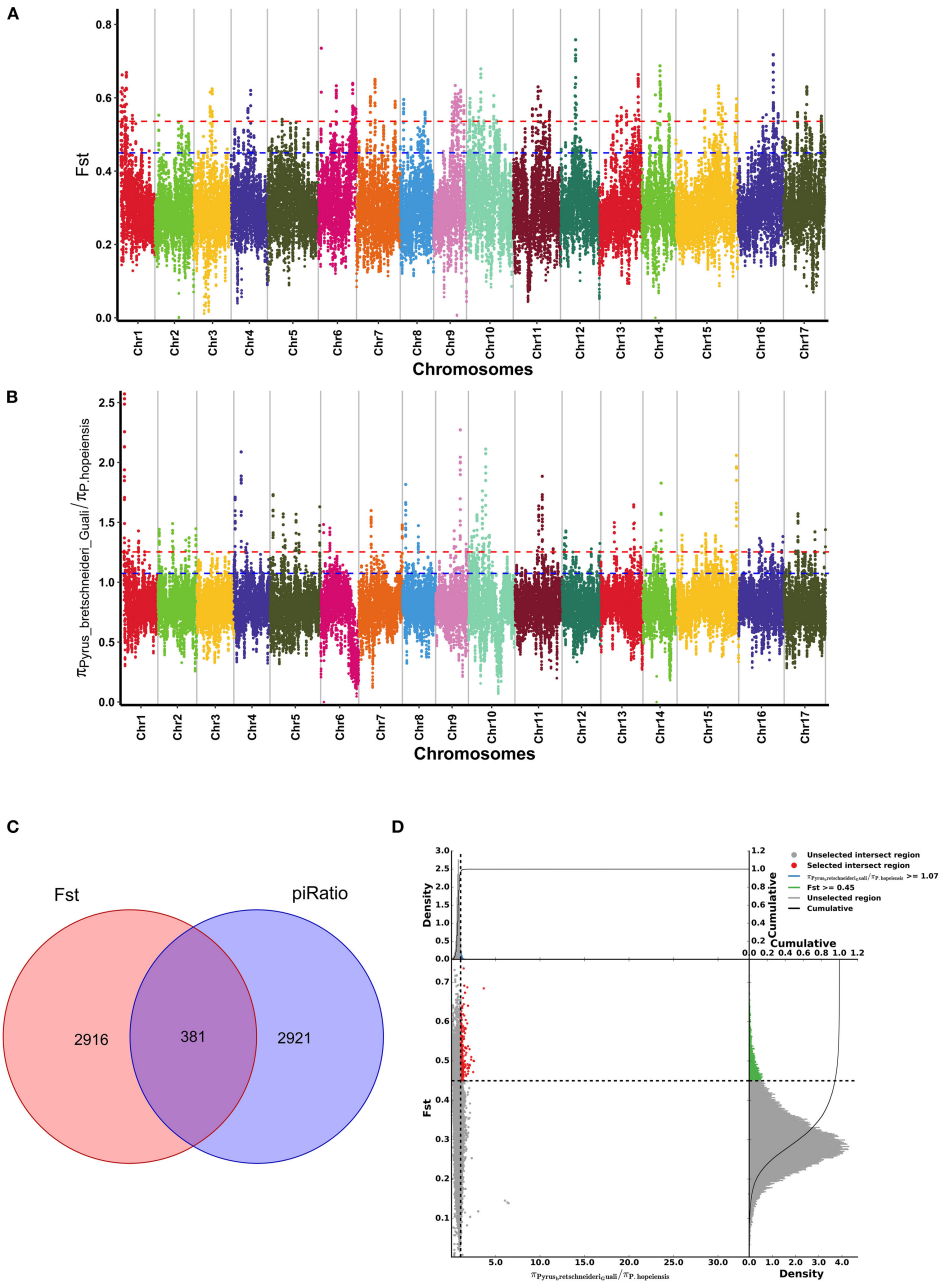
Genome-wide selection scan of *Pyrus hopeiensis*. **(A,B)** Manhattan plot of the genome-wide distribution of Fst values and π ratios in *P. hopeiensis* using a 100-kb window size and a 10-kb step size. **(C)** Venn diagram showing the gene overlap among Fst values and π ratios in the significant selection region. **(D)** Selective sweep analysis of *P. hopeiensis* based on Fst values and π ratios. The transverse coordinates are π ratios and the longitudinal coordinates are Fst values. The dot plot in the center presents the corresponding Fst values and π ratios in different windows. The red region indicates the top 5% of regions selected by π and Fst. The area below the black curve indicates the enriched area.

The Fst (Figure 8A) and π ratio (Figure 8B) were used to calculate the positive selection area between the *P. hopeiensis* population and the *P*. *bretschneideri* “Guali” population. The Fst value calculations obtained 2,916 positive selection genes, and 2,921 positive selection genes were detected by the π ratio. In addition, regions with significantly elevated Fst values (Fst > 0.45) and regions with elevated π ratios (π ratio > 1.07) were selected. A total of 381 overlapping genes including *SAUR72, IAA20, HSFA2*, and *RKP* genes with strong selection signals (Figure 8C) (Supplementary Table 7). The Fst values and the π ratios of the candidate region exceeded those of the whole genome site. Figure 8D showed the results of the Fst and π ratio selective elimination analysis for *P. hopeiensis*.

### Gene Functional Enrichment Analysis

**Figure 9:**
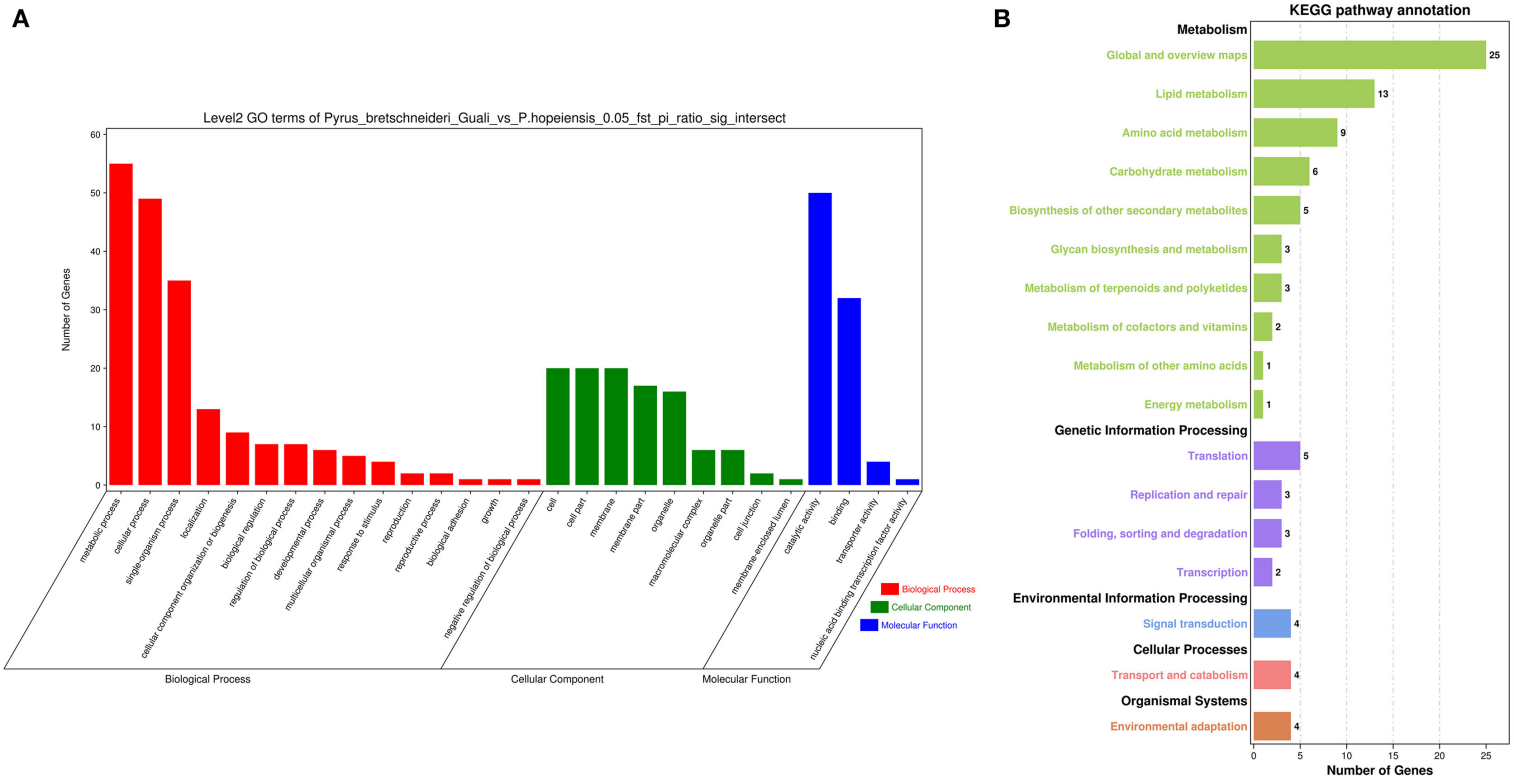
Statistical analysis of enrichment in *P. hopeiensis*. **(A)** GO analysis and **(B)** KEGG pathway analysis.

The genomic regions between *P. hopeiensis* and *P*. *bretschneideri* “Guali” were compared to identify signatures of positive selection within *P. hopeiensis* following environmental and artificial selection pressures. Gene function annotations were performed on 381 genes selected by Fst values and π ratios. The GO entries were enriched in 345 biological processes, of which 45 were significantly enriched, including glycogen metabolic process, energy reserve metabolic processes, and cellular glucan metabolic processes (Supplementary Table 8). Among the 66 enriched cell components, seven were significantly enriched, including intrinsic component of membranes, the histone acetyltransferase complex, and the protein acetyltransferase complex (Supplementary Table 9). Among the 92 enriched molecular functions, 24 were significantly enriched, including cellulose synthase activity, transition metal ion binding, and metal ion binding (Supplementary Table 10). GO Term (level 2) analysis showed that these candidate genes were enriched in 15 biological processes, nine cell components, and four molecular functions (Figure 9A; Supplementary Table 11). Metabolic process GO:0008152 had the most enriched genes (55) in the biological processes, followed by cellular process GO:0009987 with 49 genes, and single-organism process GO:0044699 with 35 genes. In the molecular function category, catalytic activity GO:0003824 had the most enriched genes (50), followed by binding GO:0005488 with 32 genes. And in the cell components category, there were 20 genes that were enriched in membrane GO: 0016020, cell GO: 0005623, and cell part GO: 0044464.

To better understand the signal pathways of candidate genes in the *P. hopeiensis* population, KEGG pathway enrichment analysis were performed on the selected candidate genes. In total, 54 pathways were enriched in the *P. hopeiensis* population (Supplementary Table 12). Among the pathways, four were significantly enriched pathways, including lysine degradation (five genes), sphingolipid metabolism (four genes), other glycan degradation (three genes), and betaine biosynthesis (two genes). These genes were classified into 17 pathways based on the annotation of KEGG class B (Figure 9B). The global and overview maps pathway had the most enriched genes (25 gene), followed by lipid metabolism (13 genes).

## Discussion

Pears originated in China, and the wild resources exist widely (Rubtsov, 1944). The protection, development and rational use of rare and endangered wild plants have become elements of the core content for protecting biodiversity. At present, the habitat conditions for *P. hopeiensis* are sub-optimal, and wild resources are being severely damaged. Changli in Hebei Province has a long history of fruit tree cultivation. Local people reclaim terrain on the nearby mountains for planting fruit trees. The local flower industry is also relatively well-developed, and the area under flower gardens has gradually expanded, invading the native habitat of *P. hopeiensis*. As the fruits of *P. hopeiensis* are small, bitter and inedible, villagers have used the nearby *P. hopeiensis* population primarily as rootstocks or they have felled the trees to expand the agricultural areas. Moreover, the *P. hopeiensis* population grew mostly on cliffs, in ravines, and in sandy areas, where their roots were largely exposed. Large numbers of climbing woody plants occurred around *P. hopeiensis* trees in the ravine, and these represent a competitive threat to the survival of the trees. Many mountainous landscapes occur in Changli and the local people have often planted *Pinus* species for greening on these mountains, and the procedure has degraded conditions required for the survival of *P. hopeiensis*. In addition, low temperatures can seriously impact the yield and distribution of pears. In recent years, temperatures have been unstable in diverse locations and pear blossoms had suffered severe freezing damage. These factors are all major threats to the expansion and survival of the *P. hopeiensis* population. If the wild resources of *P. hopeiensis* are not protected in a timely manner, the risk of extirpating the wild resources of this species will be extremely high. It is imperative to protect and restore the wild resources of *P. hopeiensis*. Thus, we have implemented protective measures for the species. These measures included intensifying publicity efforts to increase local residents' awareness of the need to protect *P. hopeiensis*, establishing *P. hopeiensis* protection communities for *in-situ* conservation, promoting scientific research on *P. hopeiensis* ecology and reproductive biology to provide basic data for *ex-situ* conservation, strengthening environmental protection in the distribution area of *P. hopeiensis*, and establishing and improving the management of *P. hopeiensis* archives.

In our study, 23 *Pyrus* accessions were re-sequenced. And the mutation types such as SNP, InDel, SV and CNV were detected and annotated. Among them, SNPs and InDels were the most abundant form of genetic variation in plant genomes (Rafalski, 2002; Montanari et al., 2019), and they were widely used in the scientific community. SNP markers were frequently used in plant genetic diversity research, the construction of high-density genetic linkage maps, and the identification of loci or genes related to complex traits (Chen et al., 2014; Winfield et al., 2016). A total of 107,133,072 SNPs and 141,866,26 insertions or deletions were detected, and most of these variants were located in intergenic regions or introns (Xanthopoulou et al., 2019; Li et al., 2020). The average dN/dS ratio of these *Pyrus* accessions was 1.044, and the numbers of synonymous SNPs exceeded the number of non-synonymous SNPs, similar to previous studies (Cheng et al., 2019; Li et al., 2019; Cui et al., 2020). However, the ratio was lower than those reported for wild soybean (1.36) (Lam et al., 2010), sweet cherry (1.78) (Xanthopoulou et al., 2020) or grapevine (1.17) (Liang et al., 2019). In addition, the heterozygous rates of different *Pyrus* species were varied, ranging from 0.19 to 0.53%, and averaging of 0.44%. The heterozygous rate of these *Pyrus* species was much lower than those of *P*. *betulifolia* (1.54%) (Dong et al., 2020), (*P*. *ussuriensis* × *communis*) × spp. “Zhongai1” (1.45%) (Ou et al., 2019) and *P*. *bretschneideri* “Dangshangsu” (1.02%) (Wu et al., 2013).

In the period of 2016–2018, many field surveys about the numbers and distribution of *P. hopeiensis* trees during different periods of flowering and fruiting were carried out. More than 100 specimens were found in the hills area near Xingshuyuan Village, and most of those in woodlands, farmlands or bordering other facilities were scattered bushes. Most specimens were shrubs produced by roots sprouting and only 10 full trees were found. The maximum age of the trees was about 50 years. Besides, five strains of *P. hopeiensis* found on Changyushan were identified using SSR molecular markers and no differences among them were found. However, there were differences in morphology and SSR markers between *P. hopeiensis* specimens from Xingshuyuan and Changyushan. Therefore, we grouped specimens in the population occurring in Xingshuyuan within the category *P. hopeiensis* HB-1, and the population on Changyushan within the category *P. hopeiensis* HB-2. In 2019, the wild pears collected in the region where we found *P. hopeiensis* were used for whole genome resequencing and the SNPs obtained were used for population structure analysis. The phylogenetic tree and the PCA analysis were congruent, indicating that *P. hopeiensis* specimens from Xingshuyuan and Changyushan were distributed on different phylogenetic tree branches, and the two groups were belonging to different genotypes. Moreover, the results showed that PWH06, PWH07, PWH09, PWH10, PWH13 were closely associated with *P. hopeiensis* HB-1, while PWH17 was more closely associated with *P. hopeiensis* HB-2. Based on this information and details of tree morphology, it speculated that *P. hopeiensis* may be a hybrid population, which was consistent with the opinion of Yu (1979), who suggested that *P. hopeiensis* is a natural hybrid secondary species. The population structure analysis showed that when *K* = 3, there were genes penetration between *P*. *bretschneideri* “Guali” (PWH01, PWH03), *P*. *betulifolia*rs (PWH12, PWH21), PWH09, PWH14, PWH17, *P*. *ussuriensis Maxin*. cv. Jingbaili (PWH22), *Pyrus communis* L. cv. Early Red Comice (PWH23), and *P. hopeiensis*. These findings differed somewhat from the results of the neighbor-joining tree and PCA. However, when *K* = 5, *P. hopeiensis* and *P*. *betulifolia* could be distinguished, although gene penetration also existed. *P*. *ussuriensis Maxin*. cv. Jingbaili is among the varieties in the *P*. *ussuriensis* Maxim system. Gene penetration occurred between *P*. *ussuriensis* Maxin. cv. Jingbaili and *P. hopeiensis*, consistent with the view that *P*. *ussuriensis* Maxim may have been a participant in the hybrid origin of *P. hopeiensis*. Besides, *P. hopeiensis* also occurs in Laoshan, Shandong Province. The cluster analysis of Liang et al. (2015) showed that *P. hopeiensis* samples from Laoshan and Changli had crossed with one another. There was no direct correlation between genetic distance and the geographic location of *P. hopeiensis* populations. Gene exchange among *P. hopeiensis* populations was frequent. The results also showed that the genetic distances between *P*. *hopeiensis, P*. *ussuriensis* Maxim and *P*. *phaeocarpa* were all smaller than the distances from other *Pyrus* taxa, indicating that close relationships between these three species. *P*. *betulifoliar* and *P*. *phaeocarpa* were also close. These molecular level data support Yu's speculation about the relationship between the three species (*P*. *hopeiensis, P*. *ussuriensis* Maxim and *P*. *phaeocarpa*). The close relationship between *P*. *betulifolia* and *P*. *phaeocarpa* may account for the genetic exchange between *P. hopeiensis* and *P*. *betulifolia* that we detected. Furthermore, *P. hopeiensis* bred mainly by natural hybridization, which also provided potential for gene penetration between *P. hopeiensis* and other congeners.

Many species have experienced strong positive selection in the long process of evolution. These species exhibit selected cancellation signals. Two methods (Fst and π ratio) were used to analyze the entire genome of *P. hopeiensis* and *P*. *bretschneideri* “Guali,” and the important selected signals of *P. hopeiensis* relative to *P*. *bretschneideri* 'Guali'. A total of 381 overlapping genes including *SAUR72, IAA20, HSFA2*, and *RKP* genes with strong selected signals were detected. *AUX/IAA* (*auxin/indole-3-acetic acid*), *GH3* (*Gretchen Hagen 3*), and *SAUR* (*Small auxin up-regulated RNA*) are the early auxin-responsive gene families in plants. Among them, *SAUR* is the largest gene family and plays a critical role in response to signals such as drought, low temperature and diseases (Ren and Gray, 2015). Low temperature stress can increase the expression of six *SAUR* genes (*PtSAUR12, PtSAUR34, PtSAUR54, PtSAUR67, PtSAUR91*, and *PtSAUR97*) in poplar trees (Hu et al., 2018). HSF (Heat shock transcription factor) is an important factor in the regulation of plant stress resistance, and plays a key role in plant cold stress response, heat resistance, drought tolerance, and salt stress (Andrási et al., 2021). The overexpressed *HbHsfA1* and *HbHsfB1* may be candidates to improve cold stress tolerance of rubber trees (Deng et al., 2018). Moreover, the KEGG analysis showed that these genes were significantly enriched in four pathways, including lysine degradation, sphingolipid metabolism, other glycan degradation, and betaine biosynthesis. Among these pathways, functional studies of sphingolipid metabolism-related genes indicated that sphingolipids have important roles in plant growth, development and response to biotic and abiotic stress (Wu et al., 2015; Li et al., 2016; Zheng et al., 2018). Sphingolipids are mainly responsible for promoting the active response of plants to various adversity coercive forces, such as drought, freezing damage, and cold damage (Dunn et al., 2004), by enhancing the stability of the plant plasma membrane and the vacuolar membrane. Betaine (GB) is a N-methylamino acid and it is a type of quaternary ammonium compound. It is ubiquitous in bacteria, algae, animals and a variety of plants (Zuo, 2019). Most plants can detect the accumulation of betaine when subjected to adversity stress, and this has an important role in resistance to external environmental stress. Betaine improves salt tolerance, cold tolerance and frost resistance in plants, and reduces the damage caused by drought stress. Under drought and salt stress, betaine can be used as a molecular chaperone to protect the activity of intracellular proteins and metabolic enzymes while participating in the processes of energy metabolism and improving the efficiency of photosynthesis (Yancey et al., 1982). GB reduced the malondialdehyde (MDA) content of sweet tea leaves under drought stress, increased the quantity of osmotic adjustment substances and the relative water content in sweet tea and it also regulated antioxidant enzyme activity (Li et al., 2017). Moreover, betaine increased the abilities of corn (Zhao et al., 2018), pomegranate fruits (Molaei et al., 2021) and other species to resist low temperature stress. Therefore, combined with phenotypic, it speculated that *P. hopeiensis* may have a better ability of cold tolerance, which provided a basis for the future research.

## Conclusions

A preliminary investigation of the distribution, numbers of survivors, habitat, and reproductive methods on *P. hopeiensis* population were first conducted. The number of *P. hopeiensis* specimens was low and the distribution was limited. More than 100 *P. hopeiensis* HB-1 individuals were found in Xingshuyuan village, and five *P. hopeiensis* HB-2 individuals were found in Changyushan village. The habitat of *P. hopeiensis* was degraded and *P. hopeiensis* were found mostly on cliffs with bare roots. It reproduced mainly by natural hybridization and by root tiller production under natural conditions. The whole genome re-sequencing of 23 *Pyrus* accessions were performed and a lot of mutations of SNPs, InDels, CNVs, and SVs were detected. The SNP and InDel mutation data are valuable resources for studying species evolution, domestication and trait discovery. Using a combination of morphological data for *Pyrus* species and the population structure constructed by SNPs, it suggested that *P. hopeiensis* was a natural hybrid secondary species, consistent with previous studies. Because the natural hybridization under natural conditions was a major means of propagation in *P. hopeiensis*, there existed a wide range of gene exchanges between *P. hopeiensis* and local *Pyrus* species. The positive selection region of *P. hopeiensis* and the functional enrichment of these positive selection genes were analyzed. And the KEGG significant enrichment analysis showed that those genes that were subject to positive selection were annotated to pathways that played an important role in plant resistance to external environmental stress, including sphingolipid metabolism and betaine. This study had implications for the evolution and classification of *P. hopeiensis* and provided a foundation for breeders aiming to develop improved pear varieties with good phenotypic traits and increased productivity using the abundant germplasm resources.

## Data Availability Statement

The datasets presented in this study can be found in online repositories. The names of the repository/repositories and accession number(s) can be found at: NCBI SRA BioProject, accession no: PRJNA724820.

## Author Contributions

MY conceived and designed the experiments and revised the manuscript. YL, SW, and YZ collected the samples and analyzed the sequence data. YL and JZ drafted the manuscript. All authors read and approved the final manuscript.

## Conflict of Interest

The authors declare that the research was conducted in the absence of any commercial or financial relationships that could be construed as a potential conflict of interest.
