# Supplementary material for: The Distribution and Origins of Pyrus hopeiensis-“Wild Plant With Tiny Population” Using Whole Genome Resequencing
Source: Front Plant Sci. 2021 Jun 17;12:668796. doi: 10.3389/fpls.2021.668796 (PMC8250157; doi:10.3389/fpls.2021.668796)
Supplement: Supplementary file 1 [file Data_Sheet_1.zip › Tables S1-4.docx]

Table S1 The information of materials

| No. | Abbreviation | Species | [Attribute](D:/Program%20Files%20(x86)/Youdao/Dict/7.2.0.0703/resultui/dict/?keyword=attribute) |
| --- | --- | --- | --- |
| 1 | PWH01 | *Pyrus bretschneideri* ‘Guali’ | wild |
| 2 | PWH02 | *Pyrus bretschneideri* ‘Guali’ | wild |
| 3 | PWH03 | *Pyrus bretschneideri* ‘Guali’ | wild |
| 4 | PWH04 | wild pear | wild |
| 5 | PWH05 | wild pear | wild |
| 6 | PWH06 | wild pear | wild |
| 7 | PWH07 | wild pear | wild |
| 8 | PWH08 | *Pyrus hopeiensis* HB-1 | wild |
| 9 | PWH09 | wild pear | wild |
| 10 | PWH10 | wild pear | wild |
| 11 | PWH11 | *Pyrus hopeiensis* HB-2 | wild |
| 12 | PWH12 | *Pyrus betulaefolia* | wild |
| 13 | PWH13 | wild pear | wild |
| 14 | PWH14 | wild pear | wild |
| 15 | PWH15 | wild pear | wild |
| 16 | PWH16 | wild pear | wild |
| 17 | PWH17 | wild pear | wild |
| 18 | PWH18 | *Pyrus hopeiensis* HB-1 | wild |
| 19 | PWH19 | *Pyrus hopeiensis* HB-1 | wild |
| 20 | PWH20 | *Pyrus hopeiensis* HB-2 | wild |
| 21 | PWH21 | *Pyrus betulaefolia* | cultivated |
| 22 | PWH22 | *Pyrus ussuriensis* Maxin. cv. Jingbaili | cultivated |
| 23 | PWH23 | *Pyrus communis* L. cv. Early Red Comice | cultivated |

Table S2 The group of cross-pollination and self-pollination

| No. | Pollination combinations | Number of pollinated flowers | Number of seeds obtained | The number of F1 |
| --- | --- | --- | --- | --- |
| 1 | No.1 *P. hopeiensis* HB-1×Huangguan | 200 | 109 | 26 |
| 2 | No.1 *P. hopeiensis* HB-1×Yali | 200 | 134 | 18 |
| 3 | No.1 *P. hopeiensis* HB-1×Weixian red pear | 200 | 79 | 25 |
| 4 | No.1 *P. hopeiensis* HB-1×Xuehua | 200 | 22 | 8 |
| 5 | No.1 *P. hopeiensis* HB-1×Dunzi li | 200 | 30 | 4 |
| 6 | No.5 *P. hopeiensis* HB-1×Huangguan | 200 | 32 | 16 |
| 7 | No.5 *P. hopeiensis* HB-1×Yali | 200 | 146 | 11 |
| 8 | No.5 *P. hopeiensis* HB-1×Weixian red pear | 200 | 236 | 26 |
| 9 | No.5 *P. hopeiensis* HB-1×Xuehua | 200 | 51 | 5 |
| 10 | No.5 *P. hopeiensis* HB-1×Dunzi li | 200 | 85 | 23 |
| 11 | Yali×*P. hopeiensis* HB-1 | 200 | 104 | 52 |
| 12 | Xianghong× *P. hopeiensis* HB-1 | 200 | 177 | 26 |
| 13 | Natural hybrid seeds of No.1 *P. hopeiensis* HB-1 |  | 143 | 42 |
| 14 | Natural hybrid seeds of No.3 *P. hopeiensis* HB-1 |  | 142 | 27 |
| 15 | Natural hybrid seeds of No.4 *P. hopeiensis* HB-1 |  | 260 | 122 |
| 16 | Natural hybrid seeds of No.5 *P. hopeiensis* HB-1 |  | 126 | 19 |
| 17 | Natural hybrid seeds of No.7 *P. hopeiensis* HB-1 |  | 87 | 18 |
| 18 | Natural hybrid seeds of No.8 *P. hopeiensis* HB-1 |  | 250 | 29 |
| 19 | Natural hybrid seeds of No.9 *P. hopeiensis* HB-1 |  | 161 | 25 |
| 20 | Natural hybrid seeds of *P. hopeiensis*HB-2 |  | 670 | 184 |
| 21 | No.1×No.1 *P. hopeiensis* HB-1 | 200 | 0 |  |
| 22 | No.1×No.5 *P. hopeiensis* HB-1 | 200 | 0 |  |
| 23 | No.1×No.12 *P. hopeiensis* HB-1 | 200 | 0 |  |
| 24 | No.1×No.13 *P. hopeiensis* HB-1 | 200 | 0 |  |
| 25 | No.5×No.1 *P. hopeiensis* HB-1 | 200 | 0 |  |
| 26 | No.5×No.5 *P. hopeiensis* HB-1 | 200 | 0 |  |
| 27 | No.5×No.12 *P. hopeiensis* HB-1 | 200 | 0 |  |
| 28 | No.5×No.13 *P. hopeiensis* HB-1 | 200 | 0 |  |
| 28 | No.12×No.1 *P. hopeiensis* HB-1 | 200 | 0 |  |
| 30 | No.12×No.5 *P. hopeiensis* HB-1 | 200 | 0 |  |
| 31 | No.12×No.12 *P. hopeiensis* HB-1 | 200 | 0 |  |
| 32 | No.12×No.13 *P. hopeiensis* HB-1 | 200 | 0 |  |
| 33 | No.13×No.1 *P. hopeiensis* HB-1 | 200 | 0 |  |
| 34 | No.13×No.5 *P. hopeiensis* HB-1 | 200 | 0 |  |
| 35 | No.13×No.12 *P. hopeiensis* HB-1 | 200 | 0 |  |
| 36 | No.13×No.13 *P. hopeiensis* HB-1 | 200 | 0 |  |

Table S3 Summary statistics of 23 *pyrus* accessions re-sequenced reads.

| Sample | Raw Base(bp) | Clean Base(bp) | Q20(%) | Q30(%) | GC Content(%) |
| --- | --- | --- | --- | --- | --- |
| PWH01 | 25024592100 | 25005534300 | 96.98 | 92.00 | 37.80 |
| PWH02 | 15967193100 | 15954034500 | 96.35 | 90.36 | 38.20 |
| PWH03 | 16961665500 | 16946511600 | 96.91 | 91.81 | 38.01 |
| PWH04 | 19648662900 | 19632771900 | 96.72 | 91.20 | 37.73 |
| PWH05 | 16996896900 | 16982428200 | 96.58 | 91.05 | 38.48 |
| PWH06 | 16418511600 | 16407795600 | 96.75 | 91.31 | 37.86 |
| PWH07 | 20229210000 | 20214066600 | 96.81 | 91.38 | 37.75 |
| PWH08 | 16336909800 | 16325675400 | 96.70 | 91.19 | 37.75 |
| PWH09 | 18878726700 | 18867639300 | 97.00 | 91.88 | 37.66 |
| PWH10 | 15610534200 | 15599518800 | 96.72 | 91.20 | 37.71 |
| PWH11 | 17247210000 | 17235938100 | 96.54 | 90.88 | 37.82 |
| PWH12 | 15283250100 | 15273820200 | 96.95 | 91.75 | 38.10 |
| PWH13 | 15857136900 | 15844820100 | 96.88 | 91.56 | 37.88 |
| PWH14 | 16061899500 | 16050389100 | 96.91 | 91.65 | 37.98 |
| PWH15 | 16913401500 | 16898826000 | 96.72 | 91.21 | 37.95 |
| PWH16 | 16675608600 | 16660190400 | 96.97 | 92.00 | 38.00 |
| PWH17 | 17842146900 | 17830909500 | 97.00 | 92.01 | 37.77 |
| PWH18 | 17314918200 | 17297811300 | 96.94 | 91.91 | 37.65 |
| PWH19 | 16330000000 | 14103000000 | 97.9% | 93.5% | 39.00 |
| PWH20 | 15769667400 | 15754716600 | 96.91 | 91.70 | 38.12 |
| PWH21 | 17802000000 | 14969000000 | 97.7% | 93.1% | 39.00 |
| PWH22 | 15405000000 | 13114000000 | 97.7% | 93.2% | 39.00 |
| PWH23 | 18786000000 | 15805000000 | 97.7% | 93.1% | 39.00 |

Table S4 The statistics of sequencing and coverage depth

| Sample | Mapped reads | Total reads | Mapping rate(%) | Average depth(X) | Coverage at least 1X(%) | Coverage at least 4X(%) |
| --- | --- | --- | --- | --- | --- | --- |
| PWH01 | 160111916 | 166703562 | 96.05 | 44.85 | 89.15 | 84.59 |
| PWH02 | 101828482 | 106360230 | 95.74 | 30.77 | 88.33 | 83.02 |
| PWH03 | 108418667 | 112976744 | 95.97 | 32.22 | 88.35 | 83.18 |
| PWH04 | 125655345 | 130885146 | 96.00 | 36.48 | 88.69 | 83.78 |
| PWH05 | 107483384 | 113216188 | 94.94 | 31.76 | 88.29 | 83.05 |
| PWH06 | 104754957 | 109385304 | 95.77 | 31.30 | 88.51 | 83.29 |
| PWH07 | 128080463 | 134760444 | 95.04 | 37.52 | 89.01 | 84.17 |
| PWH08 | 104567802 | 108837836 | 96.08 | 30.92 | 88.54 | 83.29 |
| PWH09 | 120561571 | 125784262 | 95.85 | 35.02 | 88.78 | 83.82 |
| PWH10 | 99925260 | 103996792 | 96.08 | 29.94 | 88.22 | 82.90 |
| PWH11 | 110201203 | 114906254 | 95.91 | 32.93 | 88.70 | 83.53 |
| PWH12 | 97645792 | 101825468 | 95.90 | 29.95 | 85.90 | 80.26 |
| PWH13 | 101045825 | 105632134 | 95.66 | 30.19 | 88.22 | 82.88 |
| PWH14 | 102578377 | 107002594 | 95.87 | 31.38 | 87.29 | 82.01 |
| PWH15 | 106678746 | 112658840 | 94.69 | 31.90 | 88.04 | 82.87 |
| PWH16 | 103017315 | 111067936 | 92.75 | 31.32 | 87.48 | 82.19 |
| PWH17 | 113928416 | 118872730 | 95.84 | 33.68 | 88.25 | 83.11 |
| PWH18 | 110680085 | 115318742 | 95.98 | 32.96 | 88.67 | 83.66 |
| PWH19 | 93261885 | 98518614 | 94.66 | 27.52 | 88.04 | 79.58 |
| PWH20 | 100115951 | 105031444 | 95.32 | 29.99 | 88.44 | 83.09 |
| PWH21 | 98696233 | 105292366 | 93.74 | 28.92 | 85.95 | 77.31 |
| PWH22 | 87195525 | 91732566 | 95.05 | 26.56 | 87.45 | 78.38 |
| PWH23 | 108416363 | 110460114 | 98.15 | 28.36 | 97.74 | 93.46 |
