# Supplementary figures and images for: The Distribution and Origins of Pyrus hopeiensis-“Wild Plant With Tiny Population” Using Whole Genome Resequencing
Source: Front Plant Sci. 2021 Jun 17;12:668796. doi: 10.3389/fpls.2021.668796 (PMC8250157; doi:10.3389/fpls.2021.668796)

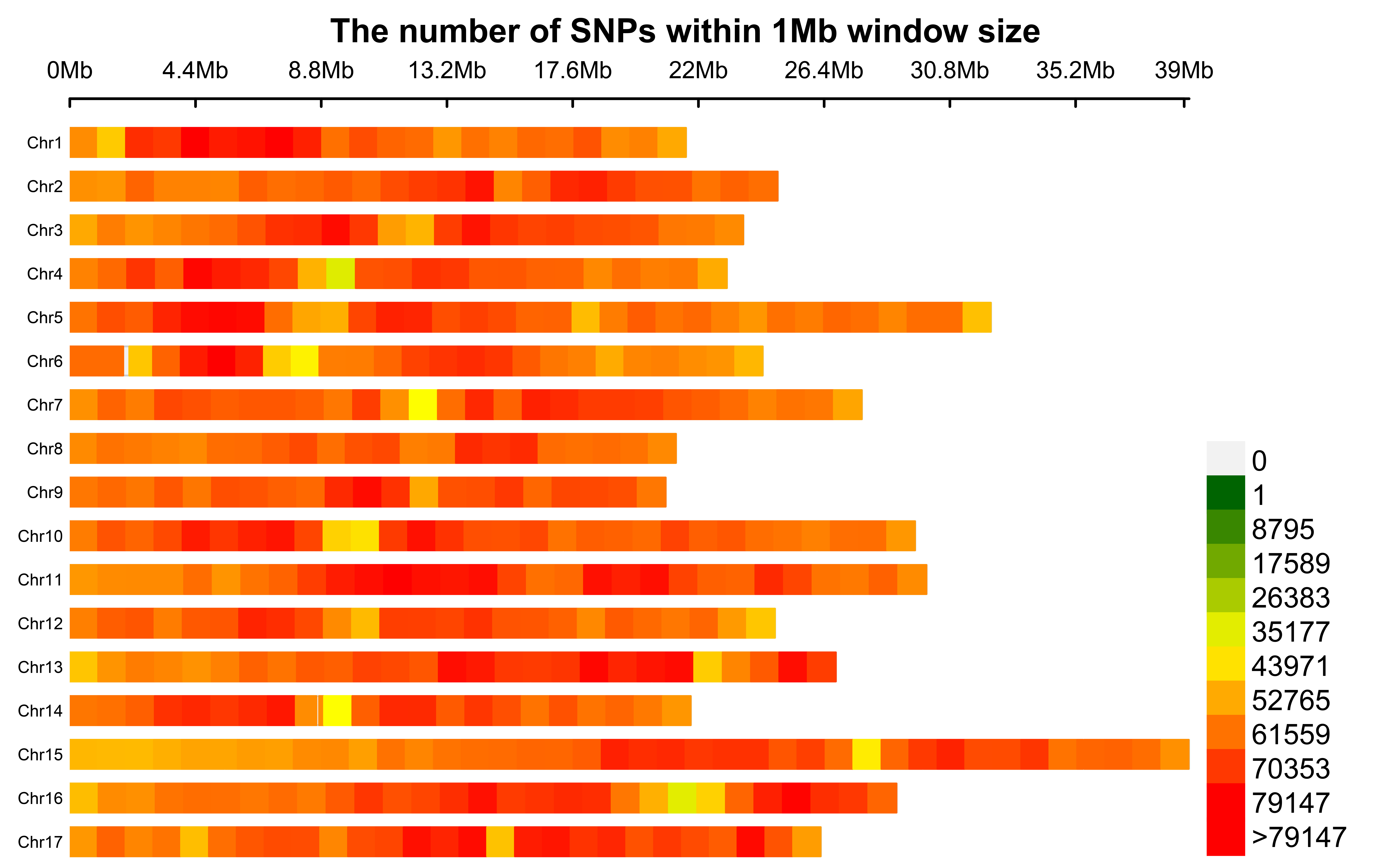

Supplement: Supplementary file 1 [file Data_Sheet_1.zip › Figure S1.jpg]

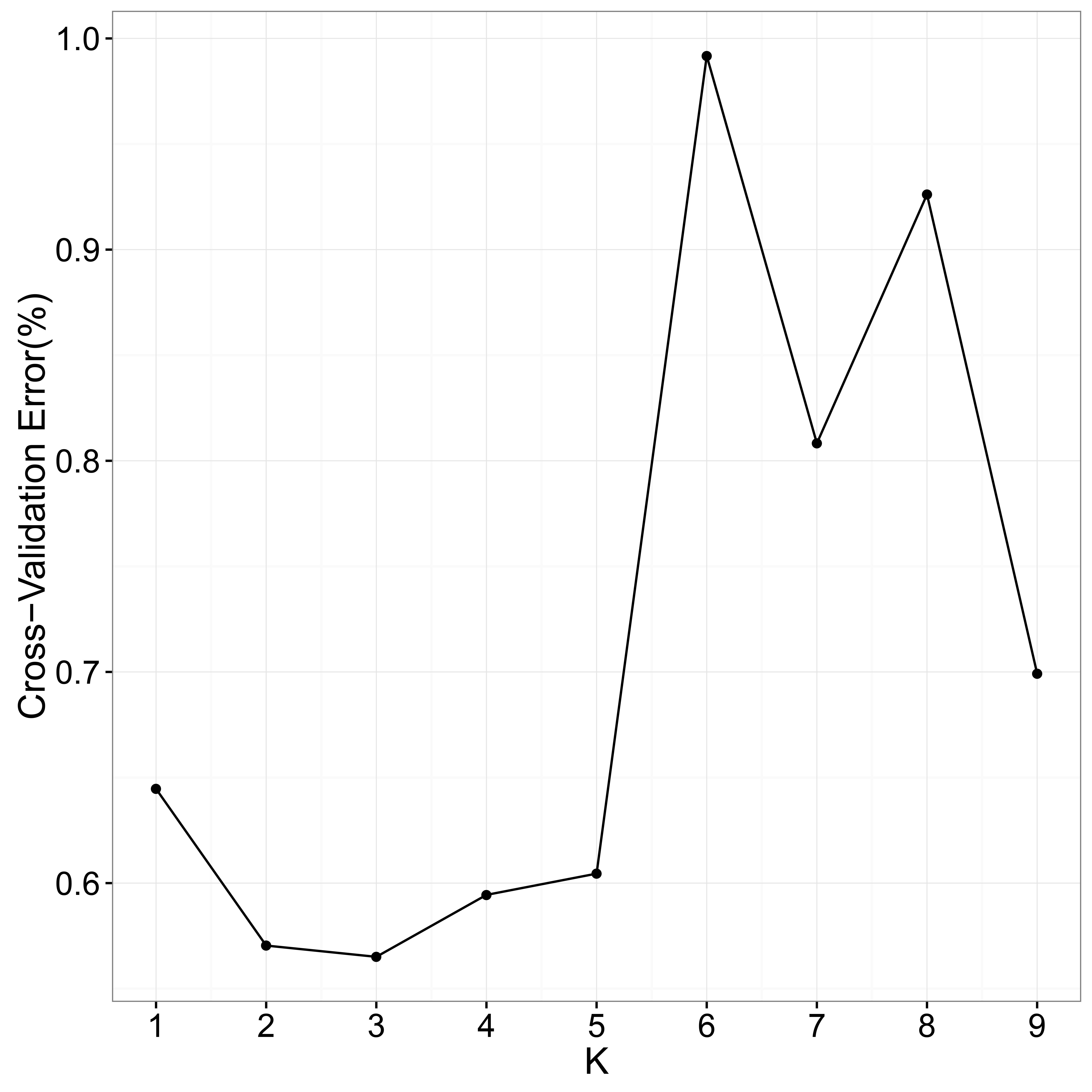

Supplement: Supplementary file 1 [file Data_Sheet_1.zip › Figure S2.jpg]

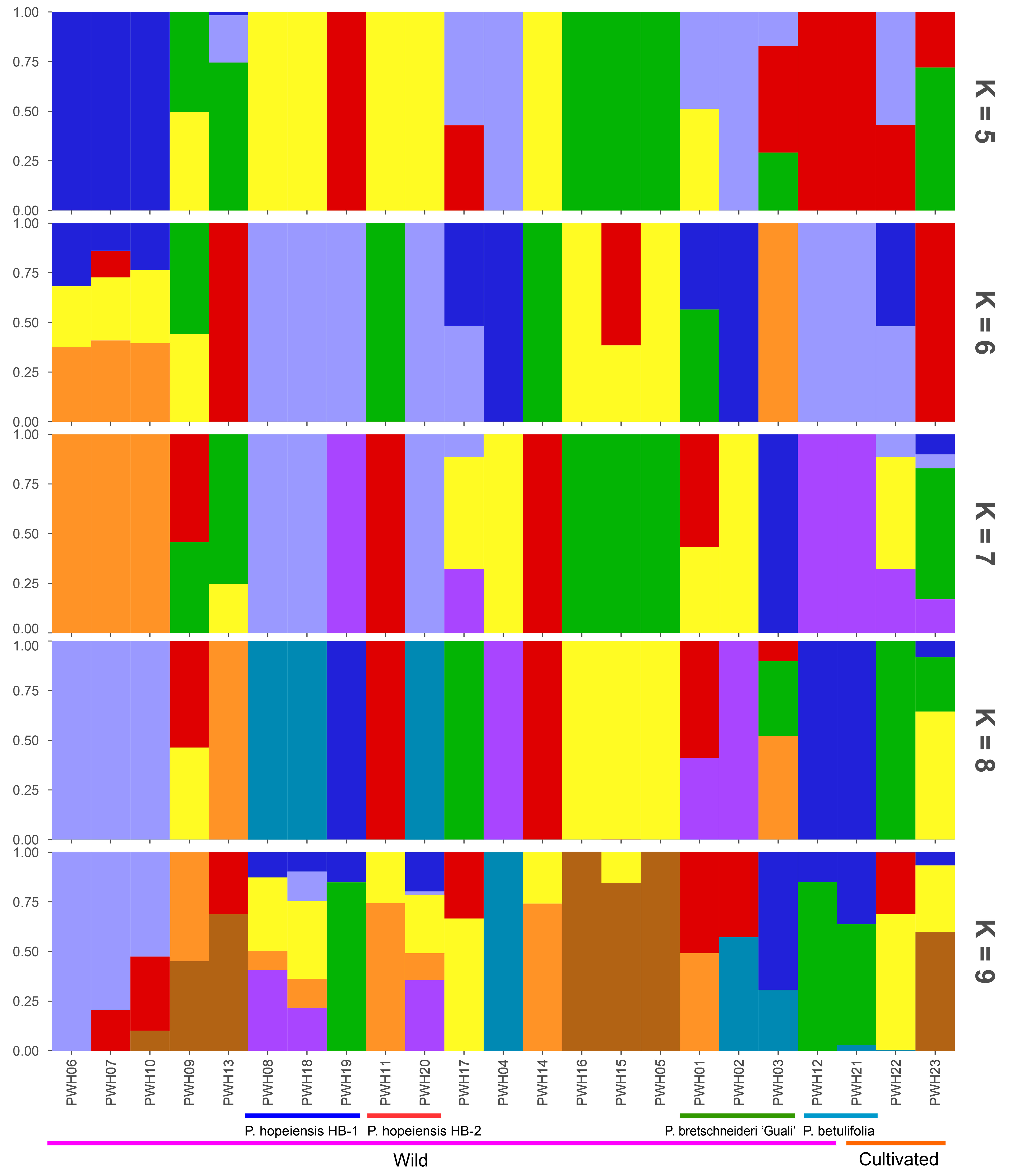

Supplement: Supplementary file 1 [file Data_Sheet_1.zip › Figure S3.jpg]

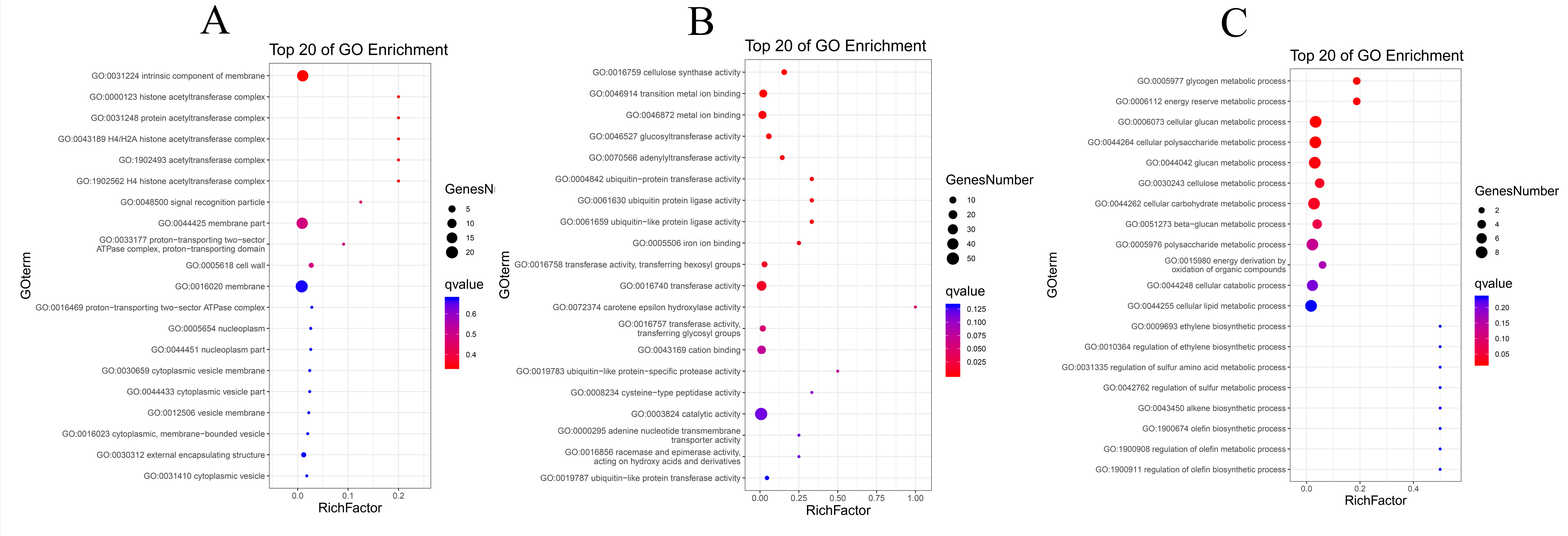

Supplement: Supplementary file 1 [file Data_Sheet_1.zip › Figure S4.jpg]
